# Variable innate lymphoid cells predominancy in oral lichen planus latently led to diverse clinical outcomes: a proof-of-concept study

**DOI:** 10.3389/fimmu.2025.1551311

**Published:** 2025-04-28

**Authors:** Xi-ye Li, Lei Pan, Yi-wen Deng, Jun-jun Chen, Zhen Tian, Guo-yao Tang, Shu-yun Ge, Yu-feng Wang

**Affiliations:** ^1^ Department of Oral Medicine, Shanghai Ninth People’s Hospital, Shanghai Jiao Tong University School of Medicine, Shanghai, China; ^2^ National Center for Stomatology, National Clinical Research Center for Oral Diseases, Shanghai, China; ^3^ College of Stomatology, Shanghai Jiao Tong University, Shanghai Key Laboratory of Stomatology, Shanghai Research Institute of Stomatology, Shanghai, China; ^4^ Department of Second Dental Center, Shanghai Ninth People’s Hospital, Shanghai Jiao Tong University School of Medicine, Shanghai, China; ^5^ Department of Oral Pathology, Shanghai Ninth People’s Hospital, Shanghai Jiao Tong University School of Medicine, Shanghai, China; ^6^ Shanghai Xin Hua Hospital, Shanghai Jiao Tong University School of Medicine, Shanghai, China

**Keywords:** innate lymphoid cells, oral lichen planus, oral lichenoid lesions, cluster analysis, cohort study

## Abstract

**Objectives:**

To search for a new classification scheme for oral lichen planus (OLP) and oral lichenoid lesions (OLL) based on innate lymphoid cells (ILCs) and to evaluate the clinical significance of this classification for diagnosis and treatment.

**Subjects and methods:**

This study was based on a clinical cohort and applied flow cytometry to prospectively analyze the ILC subgroups and proportions in OLP and OLL lesions using SPSS software (version 26.0) to attempt cluster analysis to classify diseases at the cellular level based on the phenotype and quantity of ILCs cells, analyze the correlation between the new classification of diseases and clinical risk factors based on the patient’s clinical background information and classification results, and evaluate the differences in therapeutic effects among patients in different groups in corresponding clinical cohorts.

**Results:**

In the OLP and OLL groups, the ILC compartment consisted mainly of ILC1 (75.02% ± 27.55% and 72.99% ± 25.23%, respectively), ILC2 (1.49% ± 4.12% and 1.72% ± 3.18%, respectively), and ILC3 (16.52% ± 19.47% and 18.77% ± 18.12%, respectively). Using k-means clustering and two-step clustering, patients could be clustered into three groups that did not respond equally to the same treatment. Using k-means clustering, there was a statistically significant difference in REU scores between the ILC1 advantage group and the OLL subgroup before and after treatment (*P* = 0.02), which was not observed in two-step clustering. This indicates that k-means clustering may have greater value in the clinical application of OLL. In the ILC1 absolute advantage group, using HCQ + TGP for one month could effectively treat the patients regardless of the use of k-means clustering or two-step clustering (*P* ≤0.001), whereas the other groups did not.

**Conclusions:**

This study provides a preliminary OLP and OLL classification method based on ILC subgroups that can guide the cytological classification of diseases to a certain extent. Further clinical application values should be verified in subsequent cohort studies.

## 1 Introduction

Oral lichen planus (OLP) and its relative oral lichenoid lesions (OLL) are typical interface inflammatory diseases of the oral mucosa, with a worldwide prevalence of approximately 1% (1). The malignant transformation rate of OLP/OLL can be as high as 1.14%. Given the chronic nature and high recurrence rate of these conditions, they pose a significant health burden on patients (2, 3).

OLP and OLL are widely recognized as T cell-predominant diseases (4, 5). There is a general consensus that significant immunopathological heterogeneity exists within these conditions, which could be a crucial factor leading to variability in clinical outcomes (1, 6–8). Therefore, exploring effective T-cell-based classification methods has long been a primary research focus in daily clinical work.

By combining innate and adaptive immunity, T-cell immune responses are currently classified into three types based on the innate lymphoid cells (ILCs) and T helper (Th) cell combinations (i.e., ILC1-Th1, ILC2-Th2, and ILC3-Th17). ILCs constitute a class of lymphocytes lacking antigen-specific receptors (no expression of T-cell or B-cell antigen-specific receptors), mainly consisting of ILC1, ILC2, and ILC3 subsets. They are primarily distributed at the skin and mucosal interfaces, where they play a critical role in immune surveillance and induction of T cell-mediated immunity (9–11). Recent studies have found that the proportion of total ILCs in peripheral blood was expanded in OLP and positively correlated with disease severity (12). Infiltration of various ILC subsets, especially ILC1, was also significantly increased in the OLP/OLL mucosa. Meanwhile, the ratio of ILC1/leukocytes may be beneficial for distinguishing OLP/OLL from controls (13). The above evidence preliminarily suggests that variations in ILC infiltration phenotypes and/or proportions may be applied to elucidate the immunopathological heterogeneity of OLP/OLL, potentially leading to the development of a refined classification system.

In the present study, we conducted an in-depth analysis of these inconsistently infiltrating cells within a Chinese cohort to propose a novel clinical classification framework.

## 2 Materials and methods

### 2.1 Eligibility and ethical approval

This study was reviewed and approved by the Ethics Committee of Shanghai Ninth People’s Hospital, Shanghai Jiao Tong University School of Medicine (SJTUSM), China (approval ID: SH9H-2021-T100-2, 17 May 2021). All investigators declared that this study strictly adhered to the ethical principles outlined in the Declaration of Helsinki, ensuring that the rights, safety, and well-being of participants were prioritized at all times. The study followed the principle of informed consent, providing all participants or their authorized guardians with detailed explanations of the study’s purpose, methods, potential risks, and expected benefits, and ensuring that they had sufficient time and right to make their decision. Furthermore, the privacy and personal information of participants was strictly protected, with access to relevant data restricted to authorized researchers only.

### 2.2 Participant enrollment

**Figure 1:**
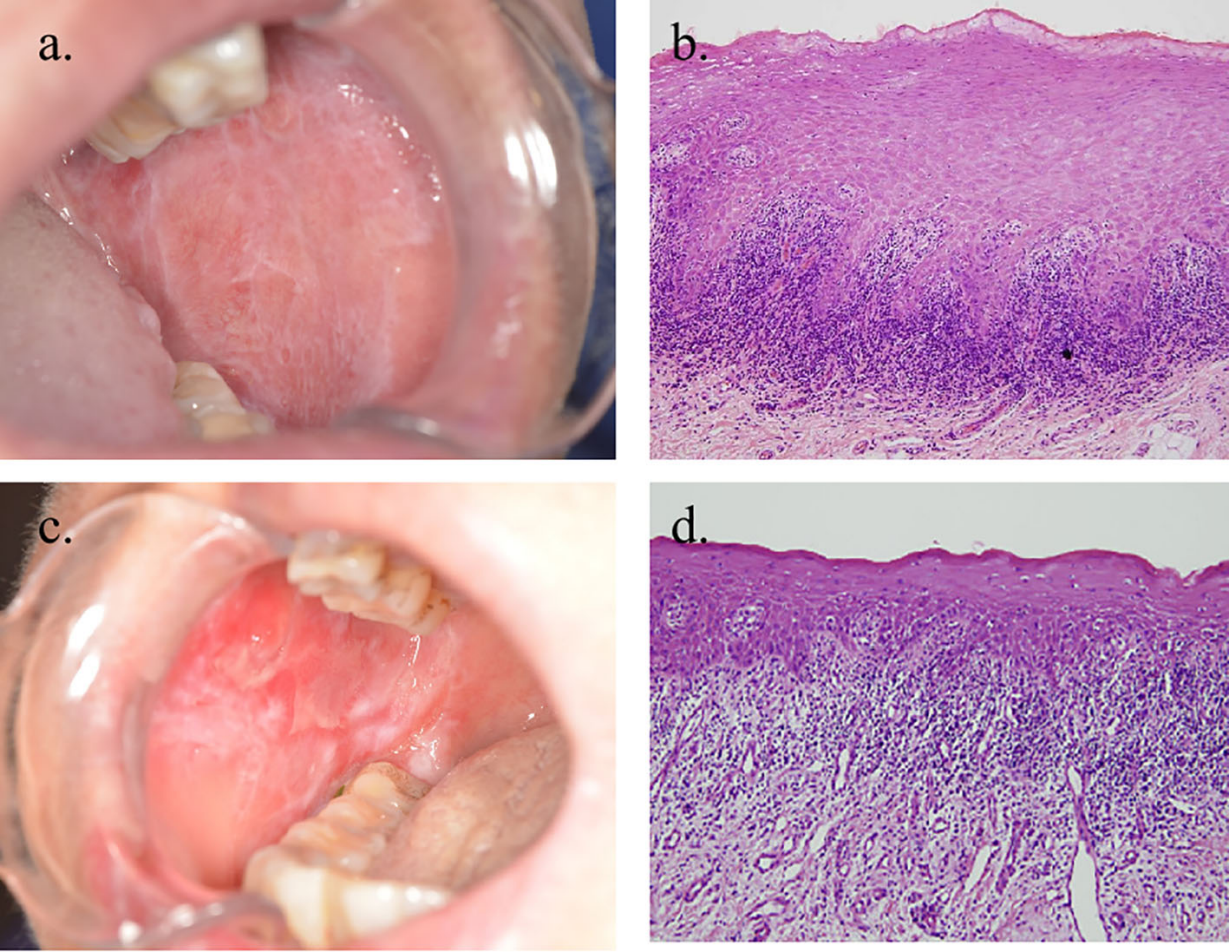
Clinical and histopathological images of OLP and OLL. **(a, b)** Clinical and microscopic images of a case in which all reviewers agreed to the diagnosis of OLP (×100 magnification). **(c, d)** Clinical and microscopic images of a patient diagnosed with OLL (×100 magnification).

Patients with OLP/OLL who visited the Department of Oral Medicine, Shanghai Ninth People’s Hospital, SJTUSM, were enrolled between 3 November 2022 and 6 December 2023. The diagnostic criteria were based on the recommendations of the American Academy of Oral and Maxillofacial Pathology in 2016 (8). Patients should have a multifocal and symmetric distribution of lesions. In addition, white and red lesions should exhibit one or more of the following forms: -reticular/popular, -atrophic (erythematous), -erosive (ulcerative), -plaque, and -bullous; lesions were not localized exclusively to the sites of smokeless tobacco placement; lesions were not localized exclusively adjacent to and in contact with dental restorations; lesion onset did not correlate with the start of a medication; and lesion onset did not correlate with the use of cinnamon-containing products. The histopathological criteria were as follows: presence of band-like or patchy, predominantly lymphocytic infiltrate in the lamina propria confined to the epithelium–lamina propria interface, basal cell liquefactive (hydropic) degeneration, lymphocytic exocytosis, absence of epithelial dysplasia, and absence of verrucous epithelial architectural change. Cases that required fulfillment of both the clinical and histopathological criteria were defined as OLP (**Figures 1a, b**). Conditions exhibiting chronic interface mucositis but otherwise failing to satisfy this set of diagnostic criteria should be designated by the clinician as oral lichenoid lesions(OLL), or the clinician should provide a descriptive diagnosis, such as “lichenoid mucositis” or “chronic mucositis with lichenoid features.” It should be noted that the patients who had the following clinical history were excluded: history of organ transplant or cGVHD; history of lupus erythematosus (systemic or discoid); history of hepatitis C and/or other virus infection liver diseases; history of using any cinnamon-containing foods or products, such as chewing gum, mints, or tartar-control toothpaste, at a time that correlates with the onset of oral lesion(s); history of use of tobacco (any form); and the onset of the oral lesions correlated with initiation of medication (**Figures 1c, d**).

The inclusion criteria were as follows (1): patients aged between 18 and 75 years (2); understanding and voluntarily providing signed informed consent; and (3) no treatment for OLP/OLL applied within the last 3 months. Healthy donors without oral inflammation or infection (including periodontitis) were enrolled as healthy controls (HCs).

The exclusion criteria were as follows (1): any pregnant women or those who had a pregnancy plan (2); any patients with severe systemic diseases that were not well controlled, including but not limited to diabetes, hypertension, heart disease, pneumonia, or any other health issues deemed by the physician to potentially affect the diagnostic or therapeutic process (3); any infection affecting the oral mucosa or other soft tissues (4); potential allergy or side effects of any medicine used for treatment (5); glaucoma, cataracts, and any other forms of retinal diseases; or (6) any mental disorders that impaired or resulted in the loss of cognitive capacity.

A preventive anti-fungal medication, 100 mg fluconazole once daily oral intake, and 1% solution of sodium bicarbonate three times daily mouthwash, was administered for 14 days prior to the pathological examination.

### 2.3 Tissue collection and single-cell sample preparation

Under local anesthesia, a punch incision (8 mm diameter, approximately 3 mm depth) was made at the lesional or healthy sites (buccal mucosa, tongue, or lip). Half of the tissue was reserved for pathological examination, and the other half was collected for single-cell sample preparation.

Each tissue was collected in 1 ml pre-cooled modified 1640 medium (Hyclone Laboratories, Logan, UT, USA; Cat.# SH30809.01) and kept at 4 °C. It was sliced into small pieces and digested with 0.25% trypsin (Genom, ZheJiang, China; Cat.# GNM25200) in a 37 °C incubator with 5% concentration of carbon-dioxide. After incubation for 15 min, the process was terminated using complete 1640 medium (with 10% fetal bovine serum). Tissue fragments were filtered using a 100-μm cell strainer (BD Biosciences, Franklin Lakes, NJ, USA; Cat. #352360). The collected cells were then centrifuged and washed two times at 800×*g*, 4 °C for 5 min. Finally, the cells were resuspended in PBS for flow cytometry.

### 2.4 Immunofluorescence examination

Paraffin-embedded sections were dewaxed and rehydrated. Heat antigen retrieval was performed using a citrate solution, followed by permeabilization with 0.1% Triton-PBS. Sections were incubated with the antibodies detailed in **Supplementary Table 1** and counterstained with 4’,6-diamidino-2-phenylindole (DAPI). The OLP/OLL sections were scanned using a Pannoramic MIDI II (3DHISTECH).

### 2.5 Flow cytometry analysis

Single-cell suspensions derived from oral tissues were stained with a panel of anti-human antibodies (**Supplementary Table 2**), which included markers for live/dead cell discrimination. Staining was performed on ice, shielded from light, and the cells were incubated for 30 min. Labeled cells were then analyzed using a BD LSR Fortessa X-20 flow cytometer (series # 01710534), and the FCM data were processed with FlowJo software (v10.0.7).

For analysis, the cells were initially gated to identify live cells that lacked lineage markers (CD3, CD11c, CD14, CD16, CD19, CD20, CD34, CD56, CD123, and FcϵRIα). Subsequently, the cells positive for CD45 and CD127 were selected as the total ILCs population for further characterization. These cells were further categorized into three distinct ILC subsets: ILC1 (CRTH2^−^CD117^−^), ILC2 (CRTH2^+^CD117^−^), and ILC3 (CRTH2^−^CD117^+^).

### 2.6 Treatment and efficacy evaluation

In this study, patients with OLP/OLL were treated with hydroxychloroquine (HCQ) (14, 15) (100 mg/tablet, administered twice daily, one tablet per dose) and Total Glucosides of Paeony Capsules (TGP) (16–18) (administered twice daily, two capsules per dose) for one month.

The Reticular, Erythematous, and Ulcerative (REU) scoring system serves as the primary measure for evaluating the severity of OLP/OLL (19). REU scores were recorded at baseline (initial visit) and one month post-treatment. A reduction of >10% in the REU score from baseline was considered indicative of treatment efficacy (20). The effectiveness of the treatment in each group was determined by the Effective Rate, calculated as follows: effective rate = (number of effective cases/total cases).

### 2.7 Statistical analysis

Student’s *t*-test (GraphPad Prism 7, GraphPad Software Inc.) was used to compare the differences between the total number of ILCs and the different ILC subsets in the OLP/OLL samples versus the oral healthy mucosa samples.

We performed consensus clustering using a hierarchical cluster algorithm (h-cluster) and determined the optimal number of subtypes. We then used the k-means and two-step clusters to obtain specific grouping information. The clustering analysis and diagrams were conducted using SPSS Statistics (version R26.0.0.0, IBM).

## 3 Results

### 3.1 Demographic and medical characteristics of controls and patients

A total of 189 samples were obtained from 29 control participants, 73 OLP patients, and 87 OLL patients (**Table 1**). The OLP group consisted of 73 patients (46 females, 27 males) with an average age of 44.05 ± 12.67 years, while the OLL group included 87 patients (61 females, 26 males) with an average age of 53.38 ± 11.78 years. The control group had 29 participants (19 females, 10 males) with an average age of 40.76 ± 15.78 years. Statistical analysis revealed no significant differences in sex distribution among the groups. No significant age differences were observed between the three groups (*P* = 0.243).

**Table 1:** Demographic information of controls and patients.

	OLP	OLL	Control
Total number	73	87	29
Gender(Female/Male)	46/27	61/26	19/10
Age range (years)	19–73	22–74	18–74
Average Age (SD)	44.05 (12.67)	53.38 (11.78)	40.76 (15.78)

### 3.2 ILCs were present in the oral mucosa of both OLP/OLL patients and controls

**Figure 2:**
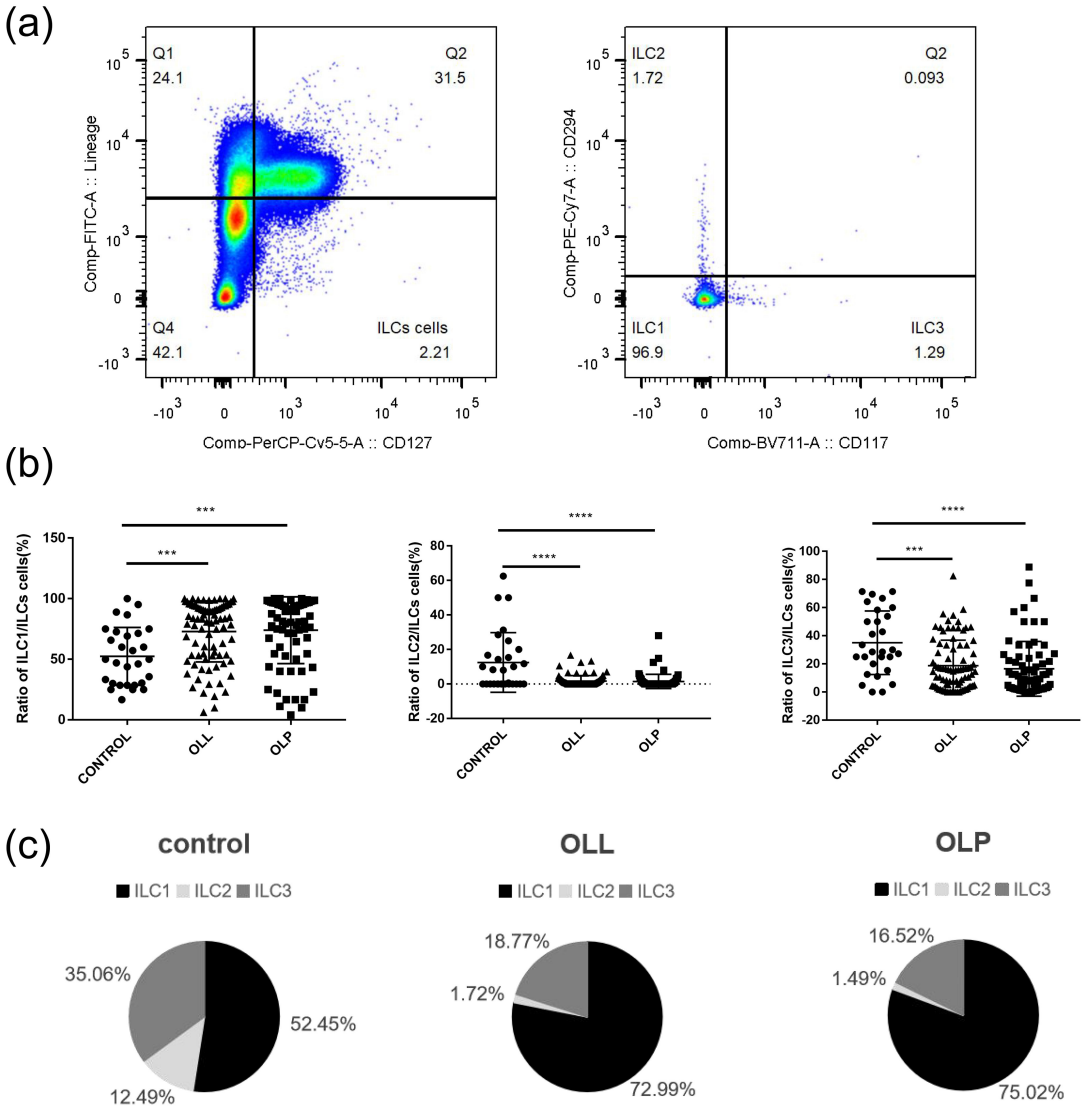
Distribution of ILC subsets in control and OLP/OLL oral mucosa. **(a)** ILCs were chosen from CD45^+^live cells, and defined as lineage (CD3, CD11c, CD14, CD16, CD19, CD20, CD34, CD56, CD123, FcϵR1α)^−^CD127^+^. The cells were then divided into ILC1 subsets (CRTH2^−^CD117^−^), ILC2 (CRTH2^+^CD117^−^), and ILC3 (CRTH2^−^CD117^+^); **(b)** the proportion of ILC1 in total ILCs statistically increased in OLP/OLL (*p* = 0.0004, *p* = 0.0002), while the proportion of ILC2 and ILC3 decreased (*p <*0.0001, *p <*0.0001; *p <*0.0001, *p* = 0.0001), compared to those in healthy controls; **(c)** the control group consisted of an average of 52.45% ILC1, 12.49% ILC2, 35.06% ILC3; the OLL group consisted of an average of 72.99% ILC1, 1.72% ILC2, 18.77% ILC3; the OLP group has an average of 75.02% ILC1, 1.49% ILC2, 16.52% ILC3. ***p <0.001;****p <0.0001.

Single cells were obtained from the oral mucosa were gated using flow cytometry analysis (**Figure 2a**).

The three percentages of ILC subsets were all significantly different between the groups, with the percentage of ILC1 in the OLP and OLL groups beingsignificantly higher than that in the control groups, and ILC2 and ILC3 rates in the OLP and OLL groups being significantly lower than those in the control groups. In the control, OLP and OLL groups, the ILC compartment consisted mainly of ILC1 (52.45 ± 23.92%, 75.02 ± 27.55%, and 72.99 ± 25.23%), ILC2 (12.49 ± 17.31%, 1.49 ± 4.12%, and 1.72 ± 3.18%) and ILC3 (35.06 ± 22.59%, 16.52 ± 19.47%, and 18.77 ± 18.12%) were also observe. It can be seen that the ILC compartment in all three groups consisted mostly of ILC1 and ILC3, while the proportion of ILC2 was the lowest. (**Figure 2c**). The proportion of ILC1 in total ILCs significantly increased in OLP/OLL (*P* = 0.0004, *P* = 0.0002), while the proportion of ILC2 and ILC3 decreased (*P <*0.0001, *P <*0.0001, *P <*0.0001, *P* = 0.0001), compared to those in healthy controls (**Figure 2b**).

**Figure 3:**
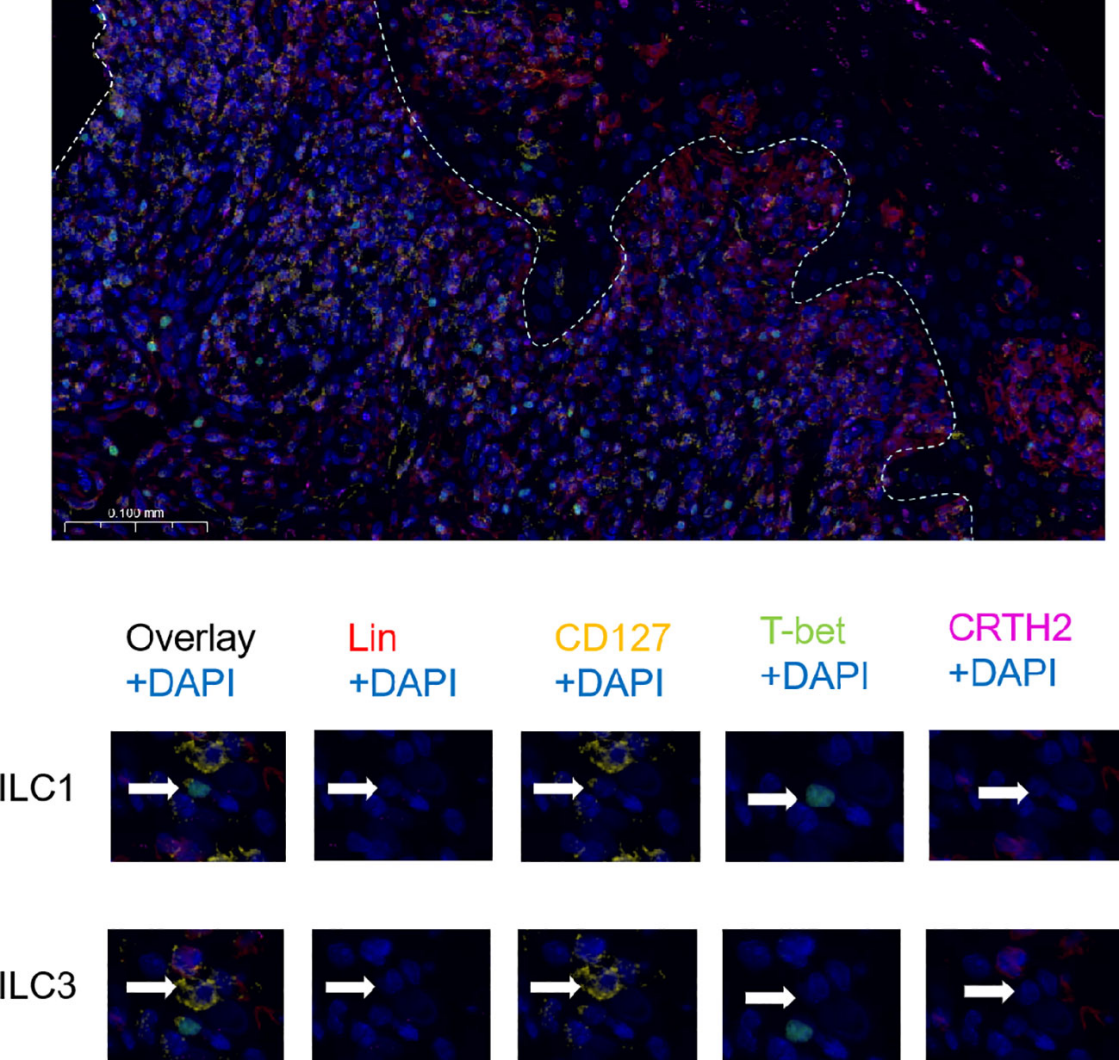
Immunofluorescence analysis of ILC subsets. The white line shows the junction between the epithelium and lamina propria. Strategy of identification of ILC subsets by immunofluorescence multicolor staining: ILCs were defined as Lin (CD3, CD14, CD20)^−^ and CD127^+^ cells and the ILC1 subset was identified as T-bet^+^, the ILC2 subset as CRTH2^+^ and ILC3 subset as T-bet^−^ and CRTH2^−^.

Immunofluorescence staining revealed the distribution of ILC subsets in the OLP oral mucosa. ILCs were defined as Lin (CD3, CD14, and CD20)^−^ and CD127^+^ cells. Hence, T-bet (nuclear) and CRTH2 (cytoplasmic) were used to distinguish the ILC1 (T-bet^+^), ILC2 (CRTH2^+^), and ILC3 (T-bet^−^ and CRTH2^−^) subsets (21) (**Figure 3**). Owing to the small number of ILC2 cells, only ILC1 and ILC3 cells are labeled in the following figure.

### 3.3 Analysis classification system clustering

In this part of the study, total 118 samples were obtained from 53 OLP patients and 65 OLL patients (**Table 2**). In the OLP group, there were 36 females and 17 males, with an age range of 19–73 years and an average age of 45.09 ± 12.65 years; In the OLL group, there were 47 females and 18 males, with an age range of 22–74 years and an average age of 54.70 ± 11.44 years. Using the chi-square test, there was no sex difference between the two groups (*P* = 0.698). Using an independent sample t-test, there was no statistically significant difference (*P* = 0.085) in age between the two groups.

**Table 2:** Demographic information of OLP/OLL patients.

Characteristic	OLP	OLL	*P-*value
Total, n (%)	53 (44.92)	65 (55.08)	
Age (years old)	45.09 ± 12.65	54.70 ± 11.44	0.08
Gender			0.70
Female	36	47	
Male	17	19	
Tobacco smoking			0.27
No	49	63	
Yes	4	2	
Hypertension			0.22
No	50	57	
Yes	3	8	
Diabetes			0.49
No	50	63	
Yes	3	2	
Hyperlipidemia			0.11
No	49	64	
Yes	4	1	
Thyroid disease			
No	49	64	0.11
Yes	4	1	

In the total, OLL and OLP groups, the proportions of ILC1 subtypes in all ILC cells were 81.92% ± 21.05%, 81.50% ± 19.46% and 84.03% ± 19.92%; The proportions of I LC2 subtypes in all ILC cells were 1.17% ± 2.62%, 1.39% ± 2.81%, and 0.90% ± 2.35%; The proportions of ILC3 subtypes in all ILC cells were 14.31% ± 18.01%, 15.48% ± 17.35%, and 13.19% ± 18.79%. It can be seen that the ILC compartment consisted mostly of ILC1 and ILC3, and the proportion of ILC2 was the lowest in the OLP and OLL groups. There was no statistically significant difference in the proportion of ILC subtypes between the OLL and OLP groups (*P >*0.05) (**Table 3**).

**Table 3:** Distributions of ILC subsets in OLP/OLL oral mucosa.

	ILC1/ILCs(%)	ILC2/ILCs(%)	ILC3/ILCs(%)
Total (n = 118)	81.92(21.05)	1.17(2.62)	14.31(18.01)
OLL (n = 65)	81.50(19.46)	1.39(2.81)	15.48(17.35)
OLP (n = 53)	84.03(19.92)	0.90(2.35)	13.19(18.79)
*P*-value	0.49	0.31	0.49

Employing unsupervised clustering analysis, we sorted different proportions of subsets of ILCs for each case. Cluster robustness was assessed by consensus clustering using h-clustering on 96 cases using three different proportions of subsets of ILCs. The optimal number of clusters (k = 3) was determined using the elbow method from the line chart, which uses the aggregation coefficients of different cluster numbers as the vertical axis and the number of clusters as the horizontal axis.

**Figure 4:**
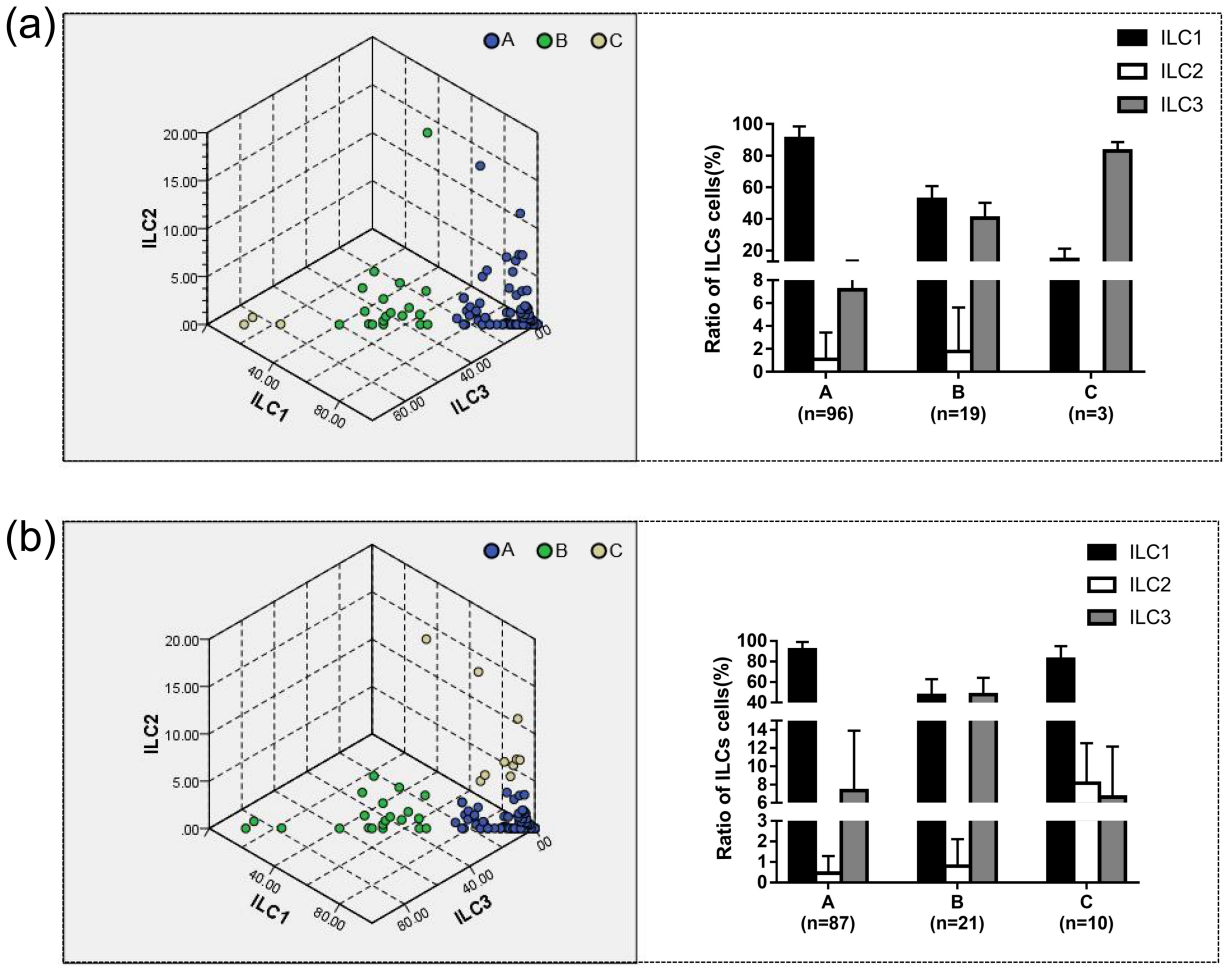
The proportion of ILC1/ILC2/ILC3 to ILC cells in different groups was determined using k-means and two-step clustering. **(a)** The results of k-means clustering showed in three-dimensional coordinates. The results showed that in Group A, the proportions of ILC1/ILCs, ILC2/ILCs, and ILC3/ILCs were 90.90% ± 7.76%, 1.09% ± 2.35%, and 7.15% ± 6.42%, respectively. Group A was defined as the ILC1^hi^ILC3^low^ (ILC1 absolute advantage) group by analyzing the characteristics of the data. In Group B, the proportions of ILC1/ILCs, ILC2/ILCs, and ILC3/ILCs were 52.20% ± 8.54%, 1.77% ± 3.85%, and 40.52% ± 9.64%, respectively. Group B was defined as the ILC1^med^ILC3^med^ (ILC3 relative advantage) group. In Group C, the proportions of ILC1/ILCs, ILC2/ILCs, and ILC3/ILCs were 14.43% ± 6.67%, 0.00% ± 0.00%, and 82.97% ± 5.61%, respectively. Group C was defined as the ILC1^low^ILC3^hi^ (ILC3 absolute advantage) group by analyzing the characteristics of the data. **(b)** Using two-step clustering, in Group A, the proportions of ILC1/ILCs, ILC2/ILCs, and ILC3/ILCs were 91.42% ± 7.74%, 0.46% ± 0.83%, and 7.32% ± 6.59%, respectively. Group A was defined as ILC1^hi^ILC3^low^ based on the characteristics of the data. In Group B, the proportions of ILC1/ILCs, ILC2/ILCs, and ILC3/ILCs were 46.91% ± 15.95%, 0.80% ± 1.31%, and 47.72% ± 16.56%, respectively. Group B was defined as the ILC1^med^ILC3^med^ group. In Group C, the proportions of ILC1/ILCs, ILC2/ILCs, and ILC3/ILCs were 82.29% ± 12.78%, 8.14% ± 4.40%, and 6.63% ± 5.52%, respectively. Group C was defined as ILC1^hi^ILC2^med^ILC3^low^ (ILC2 relative advantage) group by analyzing the characteristics of the data.

Setting the number of clusters to three, the results of k-means clustering for 118 patients as shown in **Table 4** and **Figure 4a**. In Group A proportions of ILC1/ILCs, ILC2/ILCs, and ILC3/ILCs were 90.90% ± 7.76%, 1.09% ± 2.35%, and 7.15% ± 6.42%, respectively. In Group B, the proportions of ILC1/ILCs, ILC2/ILCs, and ILC3/ILCs were 52.20% ± 8.54%, 1.77% ± 3.85%, and 40.52% ± 9.64%, respectively. In Group C, the proportions of ILC1/ILCs, ILC2/ILCs, and ILC3/ILCs were 14.43% ± 6.67%, 0.00% ± 0.00%, and 82.97% ± 5.61%, respectively.

**Table 4:** Distributions of ILC subsets in OLP/OLL oral mucosa under k-means clustering and two-step clustering.

	K-means clustering group			Two-step clustering group		
	Group A	Group B	Group C	Group A	Group B	Group C
ILC1/ILCs(%)	90.90 (7.76)	52.20 (8.54)	14.43 (6.67)	91.42 (7.74)	46.91 (15.95)	82.29 (12.78)
ILC2/ILCs(%)	1.09 (2.35)	1.77 (3.85)	.00 (.00)	0.46 (0.83)	0.80 (1.31)	8.14 (4.40)
ILC3/ILCs(%)	7.15 (6.42)	40.52 (9.64)	82.97 (5.61)	7.32 (6.59)	47.72 (16.56)	6.63 (5.52)
Number of cases	96	19	3	87	21	10

Two-step clustering automatically divided the data into three groups, and the clustering quality was good in this classification situation, which was mutually verified with the results of the system clustering. The results of the two-step cluster analysis of 118 patients are shown in **Table 4** and **Figure 4b**. In Group A proportions of ILC1/ILCs, ILC2/ILCs, and ILC3/ILCs were 91.42% ± 7.74%, 0.46% ± 0.83%, and 7.32% ± 6.59%, respectively. In Group B, the proportions of ILC1/ILCs, ILC2/ILCs, and ILC3/ILCs were 46.91% ± 15.95%, 0.80% ± 1.31%, and 47.72% ± 16.56%, respectively. In Group C, the proportions of ILC1/ILCs, ILC2/ILCs, and ILC3/ILCs were 82.29% ± 12.78%, 8.14% ± 4.40%, and 6.63% ± 5.52%, respectively.

Group A could be defined as the ILC1 absolute advantage group or ILC1^hi^ILC3^low^ group via analysis of the characteristics of the data. Group B was defined as the ILC3 relative advantage group or the ILC1^med^ILC3^med^ group. Group C could be defined as the ILC3 absolute advantage group or ILC2 relative advantage group using different clustering methods.

According to the phenotype and quantity distribution of ILCs, clustering analysis could divide patients into three groups. Using k-means clustering and two-step clustering, the group with the highest number of cases was the ILC1^hi^ILC3^low^ group, followed by the ILC1^med^ILC3^med^ group, and the group with the lowest number was the ILC2 (10 cases in two-step clustering) relative advantage or ILC3 (three cases in k-means clustering) absolute advantage group. We conjectured that the proportion of ILC3 might be the key to classification, and heterogeneity was assumed as the proportion of ILC3 in the lesions.

### 3.4 Clinical cohort studies to evaluate the effect of ILCs phenotype on OLP/OLL efficacy

Next, we assessed the effect of treatment in three different groups of OLP/OLL patients. A total of 58 OLL and OLP patients were further enrolled, including 19 males and 39 females, with an age range of 19–73 years and an average age of 47.61 ± 12.15 years old. Among them, 27 and 31 were diagnosed with OLL and OLP, respectively. The baseline average REU score was 5.41 ± 2.75.

**Figure 5:**
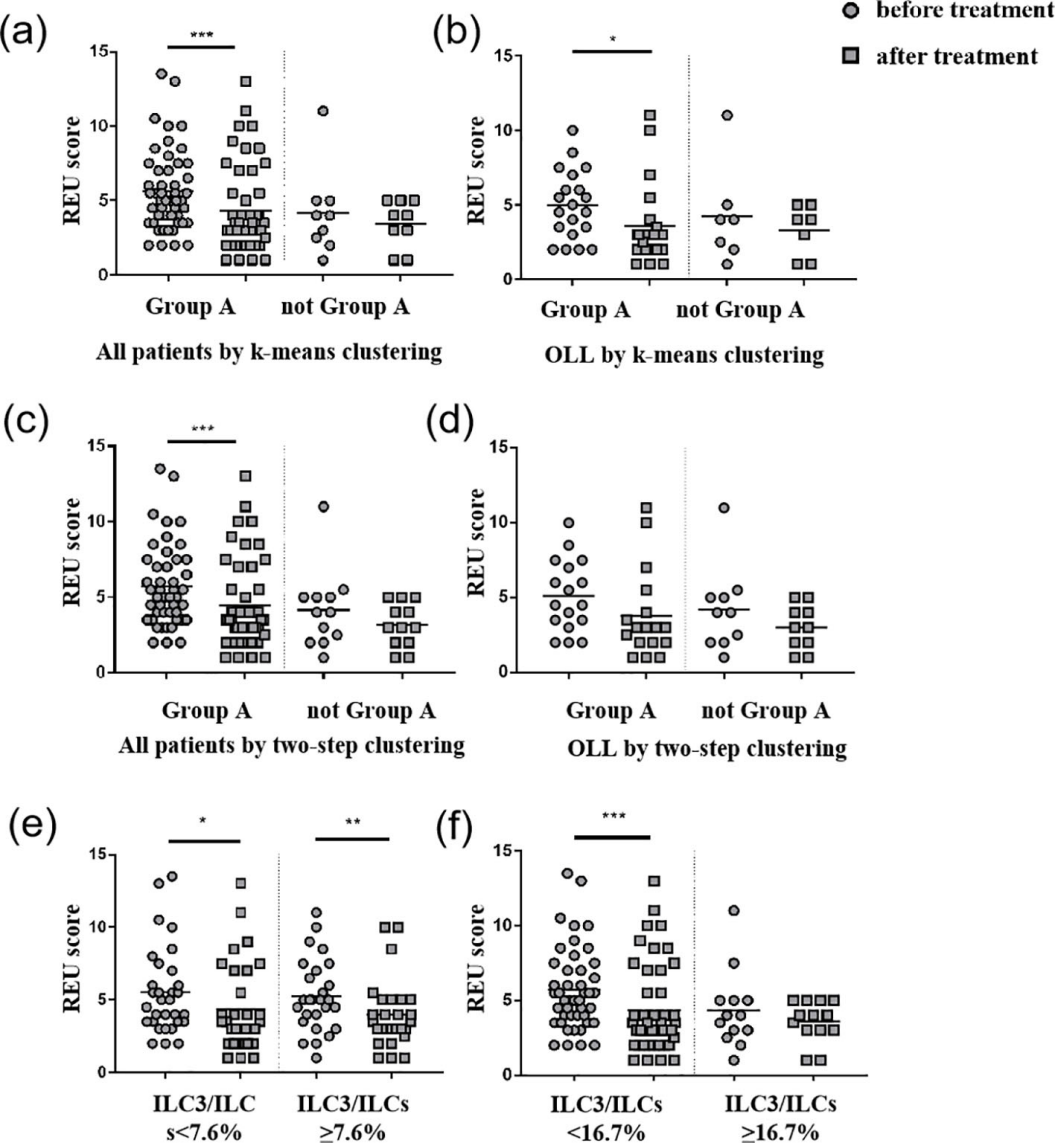
REU scores before and after treatment. **(a)** Group A (ILC1^hi^ILC3^low^) showed a statistically significant decrease (*P <*0.001) in REU scores after treatment in all patients using k-means clustering. **(b)** Group A (ILC1^hi^ILC3^low^) showed a statistically significant decrease (*P* = 0.023) in REU scores after treatment in OLL patients using k-means clustering. **(c)** Group A (ILC1^hi^ILC3^low^) showed a statistically significant decrease (*P* = 0.001) in REU scores after treatment in all patients using two-step clustering. **(d)** No group showed any statistically significant difference in REU scores after treatment in OLL patients using two-step clustering. **(e)** Patients with ILC3/ILCs <7.6% (median) and ILC3/ILCs≥7.6% showed a statistically significant decrease (*P* = 0.024; *P* = 0.002) in REU scores after treatment. **(f)** Patients with ILC3/ILCs<16.7% (third quartile) showed a statistically significant decrease (*P <*0.001) in REU scores after treatment. *p <0.05; **p <0.01; ***p <0.001.

In the case of k-means clustering, the value of efficiency displayed differences, while there was no statistically significant difference in the effective rate between the groups (**Table 5**). The ILC1^hi^ILC3^low^ (ILC1 absolute advantage) group (n = 49) showed a statistically significant difference (*P <*0.001) in REU scores before and after treatment, while the ILC1^med^ILC3^med^ (ILC3 relative advantage) group (n = 8) did not (**Figure 5a**). In the OLL subgroup, the ILC1^hi^ILC3^low^ group (n = 20) showed a statistically significant difference (*P* = 0.023) in REU scores after treatment, while the ILC1^med^ILC3^med^ group (n = 7) did not (**Figure 5b**).

**Table 5:** Baseline of 58 patients under k-means clustering and two-step clustering.

	K-means clustering				Two-step clustering			
	Group A	Group B	Group C	*P-*value	Group A	Group B	Group C	*P*-value
Total number	49	8	1		46	8	4	
Male/Female (case)	16/33	3/5	0/1	NS	15/31	2/6	2/2	NS
Age (year)	47.96 ± 12.13	42.88 ± 14.79	46	NS	47.57 ± 12.31	43.75 ± 14.73	50.25 ± 9.84	NS
REU score before treatment	5.63 ± 2.69	4.06 ± 3.07	5	NS	5.73 ± 2.72	4.44 ± 2.97	3.63 ± 1.89	NS
REU score after treatment	4.33 ± 2.86	3.25 ± 1.58	5	NS	4.46 ± 2.90	3.25 ± 1.58	3.00 ± 1.41	NS
D-value of REU score	1.31 ± 2.38	0.81 ± 2.56	0	NS	1.27 ± 2.43	1.19 ± 2.10	0.63 ± 2.81	NS
Effective cases (efficiency)	30 (61.22%)	3 (37.5%)	0 (0%)	NS	28 (60.87%)	3 (37.5%)	2 (50%)	NS
Adverse reactions	0	0	0	NS	0	0	0	NS

In the case of the two-step clustering, the value of efficiency displayed differences, whereas there was no statistically significant difference in the effective rate between any two groups (**Table 5**). The ILC1^hi^ILC3^low^ group (n = 46) showed a statistically significant difference (*P* = 0.001) in REU scores before and after treatment, whereas the ILC1^med^ILC3^med^ group (n = 8) and ILC1^hi^ILC2^med^ILC3^low^ (ILC2 relative advantage) (n = 4) did not (**Figure 5c**). In the OLL subgroup, no statistically significant differences were observed in any of three groups (*P >*0.05) (**Figure 5d**).

We also compared the REU scores in patients with ILC3/ILCs <7.6% (median) and ILC3/ILCs ≥7.6%, which all showed a statistically significant decrease (*P* = 0.024; *P* = 0.002) after treatment. Patients with ILC3/ILCs <16.7% (the third quartile) showed a statistically significant decrease (*P <*0.001) in REU scores after treatment, while patients with ILC3/ILCs <16.7% did not. The D- values of REU scores and efficiency between the groups did not show any statistically significant differences. Therefore, 16.7% (third quartile) may be a better cutoff value than 7.6% (median) in this study (**Figures 5e, f**).

## 4 Discussion

Our results showed that ILCs were present in healthy individuals and patients with OLP/OLL. The infiltration of ILC1 in the OLP/OLL mucosa increased significantly, whereas that of ILC2 and ILC3 decreased. Considering the ILCs infiltration pattern, there was no significant difference between OLP and OLL. Based on the phenotype and distribution of ILCs, cluster analysis divides patients into three groups that do not respond equally to the same treatment. This study was partially consistent with the findings in the peripheral blood of OLP (12), which found that the proportion of total ILCs was expanded and a markedly elevated ILC1/ILC2 ratio in OLP. Meanwhile, ILC3 were also increased in lesional tissues in our study, which indicated that ILC3 may play a role in the oral mucosa.

ILC1s monitor the immune system and defend against the host. ILC1s are developmentally reliant on T-bet and may contribute to Th1 cell response (22). ILC1s are the first primary producers of IFN-γ *in vivo* during the early stages of viral infection, thereby limiting viral replication at the initial site of infection (23). ILC3 depend on the transcription factor RORγt. They secrete IL-22 and certain subsets can produce IL-17A in response to IL-23 and IL-1β (24). Evidence indicates that ILC3s play a pathogenic role in atopic dermatitis through the secretion of IL-17A and IL-22 (25). Based on previous studies and the role of ILCs in the immune network, it is likely that the increased ILC1 and ILC3 in OLP/OLL tissues function by secreting cytokines involved in recasting the regional immune microenvironment and promoting the differentiation and activation of T cells in the local environment, resulting in an inflammatory state in the mucosa.

ILC2s are highly diverse and play crucial roles in regulating tissue homeostasis and repair. ILC2s can also regulate the functions of other type 2 immune cells, including Th 2 cells, type 2 macrophages, and eosinophils (26). Inflammation in psoriasis-like mice was suggested to be mediated by the IL-23-driven conversion of homeostatic skin ILC2s into a pathogenic ILC3-like state. In the oral mucosa, the conversion of inflammatory ILC2s to ILC3s is involved in protection against fungal infections (27). ILC2 are also involved in the repair of skin damage (9). Deficiency of ILC2 may lead to obstacles in the repair process of oral mucosal injury, resulting in prolonged inflammation.

The existing definitions of OLP and OLL are based on clinical and histological manifestations but without specific boundaries between them (8). According to the diagnostic criteria proposed by American Academy of Oral and Maxillofacial Pathology in 2016, compared to the clear diagnostic criteria of OLP, “Conditions exhibiting chronic interface mucositis but otherwise failing to satisfy this set of diagnostic criteria should be designated by the clinician as oral lichenoid lesions(OLL).” As can be seen before, there is a certain degree of uncertainty in the diagnostic criteria for OLL. Owing to the lack of immunopathological criteria, it is objectively impossible to clearly distinguish diseases with different immunopathological mechanisms but similar clinical and pathological manifestations, which are all classified as OLL. The traditional classification method of OLL relies on risk factors in the medical history, such as the presence of restorations and the use of suspicious drugs. OLL with mossy clinical manifestations can be further classified into oral lichenoid contact lesions, oral lichenoid drug reactions and other types (6, 7). The existing classification system still cannot effectively guide pathological diagnosis and clinical treatment, especially for OLL cases caused by unclear or multiple risk factors. In addition, for cases that meet the diagnosis of OLL but do not have the aforementioned risk factors, this classification method cannot effectively distinguish them, which leads to significant uncertainty and prognostic differences in the clinical diagnosis and treatment of OLL.

As we all know, both OLP and OLL are widely considered a T cell predominant immune reaction along the epithelium interface (4, 5). However, significant differences exist in the distribution and composition of infiltrating immune cells, leading to obvious heterogeneity in immunopathology. Recently, there has been evidence that B cells are present in the OLP (8, 28). In a study of lymphoid follicular-like structures in the oral mucosa, it was also found that CD20^+^B cells were widely infiltrated in OLP (29, 30). Previous studies have found that the composition of infiltrating immune cells in OLP is significant heterogeneous. In addition to the T cell-mediated cellular immune response, B cells, plasma cells, mast cells, granulocytes, macrophages, and innate lymphoid cells could also be found in the lesions. The subtypes of T helper cells also vary with the disease (28).

Meanwhile, there were differences in the immune cell infiltration patterns between the OLP and OLL lesions. Based on the same or similar diagnostic criteria, some authors evaluated and compared the clinicopathological features of OLP and OLL (31–33). In OLL, although inflammatory cells are more diverse, T-cell immunity is also considered an essential player (6, 34). Histopathologically, more eosinophils, plasma cells, granulocytes, and Langerhans cells have been observed in OLL lesions than in OLP lesions (31–36).

From this, it could be seen that there is clear heterogeneity in the local immune response types of OLP and OLL lesions, and there is currently no clear scheme for classifying this immune heterogeneity in clinical applications. In this study, we conducted a preliminary exploration in this direction.

We attempted to classify OLP and OLL from the perspective of immunopathology, search for differences in efficacy between different categories, and help diagnose and treat them more precisely. This study found that there may not be any difference between OLP/OLL based on ILCs, indicating that the classification of OLP and OLL concepts, generally, had not immunopathological differences. In contrast, within the OLP/OLL lesions, immune cell infiltration patterns showed significant differences. Thus, the immunopathological heterogeneity of OLP/OLL could be analyzed by proper methods based on the phenotypes and ratio of infiltrating immune cells.

The present study used clustering analysis to propose new classification hypotheses for diseases based on ILC subsets proportion and established a clinical cohort for preliminary validation, preliminarily demonstrating the significance of classifying diseases based on the immunopathological manifestations of ILC subpopulation distribution for diagnosis and treatment.

Cluster analysis is a multivariate statistical analysis method that classifies individuals or samples based on their characteristics so that individuals within the same category have the highest possible homogeneity, whereas individuals in different categories have the highest possible heterogeneity. First, we applied H-clustering to determine the number of classification categories. K-means clustering and two-step clustering were then used to compare whether the classification was consistent. K-means clustering can only classify continuous variables and requires manual setting of the number of classification categories. Two-step clustering can cluster both continuous and categorical variables simultaneously, and automatically determine the appropriate number of classifications. In this study, all three clustering methods were applicable, and clustering grouping was based on the proportion of the three ILC subgroups to the total number of ILC cells. In this study, k-means clustering and second-order clustering were used to obtain grouping hypotheses. A clinical cohort was established to compare the difference in efficacy before and after treatment between the two groups, as well as between groups, to verify the classification hypotheses and attempt to find a more clinically meaningful classification method.

Although different clustering methods had a certain impact on the number of final groups, the data characteristics of the groups were similar and distinct. In each method, the proportion of the ILC1 subset was significantly predominant in total ILC cells, indicating that ILC1 and its downstreaming Th1-cells (ILC1-Th1) immune response was widespread and dominant in OLP/OLL. Meanwhile, the critical heterogeneity may be related to the proportion and participation of ILC3-Th17 cells. Traditionally, Th17 cells are considered the primary source of IL-17; however, recent studies have shown that IL-17 produced by ILC3s potentially plays an important role in skin inflammation (37). Advances in the pathogenesis of OLP/OLL suggest that the presence of Th17 cells and the upregulation of IL-17 expression are critical (38). IL-17 overexpression was found in the lesional tissues and serum of OLP/OLL patients and was positively correlated with the severity of the disease (38). CD4^+^IL-17^+^ and CD8^+^IL-17^+^ lymphocytes are commonly found infiltrating the dying epithelial keratinocytes in OLP/OLL (39, 40). A study found that the HSP90 complex isolated from OLP lesions activated TLR9/IFN-α in DCs and further promoted the polarization of naïve T cells toward Th17 immunity (41). In dermal LP, IL-17 promotes downstream effects of the pro-inflammatory cascade by recruiting inflammatory effector cells and activating keratinocytes (42). This evidence supports our conjecture that ILC3-Th17 cells may regulate the change and progression of OLP/OLL regional lesions through IL-17. In the future, more targeted drugs (such as Th17/IL-17 antibodies) may be used to treat the ILC1^med^ILC3^med^ (ILC3 relative advantage) group, which had an unsatisfactory curative effect on conventional therapy, but a second large number of patients in our study.

Hydroxychloroquine (HCQ) inhibits intracellular and lysosomal acidification in antigen-presenting cells and suppresses pattern recognition receptors (TLR7, TLR9, and cGAS-STING) in phagocytic cells (dendritic cells, macrophages, and B cells) during innate immune response. This interference reduces antigen presentation and inflammatory mediator production (43, 44). Total glucosides of paeony (TGP), an immunomodulatory drug, regulate cytokines such as IL-1, IL-2, IL-17, TNF-α, and NF-κB. It modulates both T cell-mediated cellular immunity and B cell-mediated humoral immunity and is widely used for autoimmune diseases (45). Studies have shown that HCQ significantly reduces serum TNF-α levels and downregulates Th1 transcription factor T-bet expression (46). Similarly, TGP treatment decreased Th1 and Th17 cell populations in collagen-induced arthritis (CIA) mice, suppressing T-bet, RORγt, and STAT1/STAT3 phosphorylation (47). In psoriatic arthritis patients, a 12-week TGP regimen led to a sustained reduction in peripheral Tregs and Th1 cells, accompanied by lower Th1-type cytokines (48). Additionally, in a Sjögren’s syndrome mouse model, TGP improved disease outcomes by modulating the Th1/Th2 cytokine balance and reducing IFN-γ, IL-4, Fas, and FasL expression (49). Although these studies indicate that TGP and HCQ influence Th1-related transcription factors and cytokines, no direct evidence suggests that they specifically target Th1, Th2, or Th17 cells. Our findings show that HCQ + TGP is more effective in the ILC1-dominant group; however, further research is needed to clarify the underlying mechanisms.

In our cohort, considering the effective rate of the population, there was no statistically significant difference between any two of three groups. Using k-means clustering, a statistically significant difference was observed in the REU score of the ILC1^hi^ILC3^low^ (ILC1 absolute advantage) group in the OLL subgroup after treatment. However, there was no statistically significant difference in the REU scores of the ILC1^hi^ILC3^low^ group under two-step clustering, indicating that k-means clustering may be a more meaningful clustering method for clinical treatment in the OLL subgroup. Due to limitations in the sample population, sample size, and observation time (50, 51), we tend to test the hypothesis in a larger and long-term cohort study in the future. Meanwhile, cluster analysis is a method of making hypotheses through statistical methods, and the specific classification category may still need to be further adjusted by setting ideal cutoff values and verifying it by clinical research. The lack of moderate-to-severe patients in this study may also be a factor in the inability to identify differences between clustering groups.

## 5 Conclusion

Our results showed that ILCs were present in healthy and OLP/OLL. Based on the phenotypes and proportions of each ILC subset, OLP/OLL lesions were clustered into three groups with diverse clinical outcomes. Therefore, our results may provide a feasible hypothesis for clinical classification, diagnosis, and treatment, which needs to be verified by subsequent cohort studies.

## Data Availability

The raw data supporting the conclusions of this article will be made available by the authors, without undue reservation.
