# Supplementary material for: Variable innate lymphoid cells predominancy in oral lichen planus latently led to diverse clinical outcomes: a proof-of-concept study
Source: Front Immunol. 2025 Apr 28;16:1551311. doi: 10.3389/fimmu.2025.1551311 (PMC12066506; doi:10.3389/fimmu.2025.1551311)
Supplement: Supplementary file 1 [file Table1.docx]

Supplementary Material

# Supplementary Figures and Tables

## Supplementary Tables

**Supplementary Table 1. Antibodies used for immunoflurescence**

| Marker | Manufacturer | Category No. |
| --- | --- | --- |
| Lineage* |  |  |
| CD3 | Abcam | Ab16669 |
| CD14 | Abcam | Ab183322 |
| CD20 | Cst | 48750 |
| CD127 | Abcam | Ab118527 |
| T-bet | Abcam | Ab150440 |
| CRTH2 | proteintech | 25264-1-AP |

^*^: Lineage is composed of antibodies for CD3, CD14, and CD20.

**Supplementary Table 2. Antibodies used for flow-cytometry**

| Manufacturer | Category No. | Marker | Fluorescence |
| --- | --- | --- | --- |
| Biolegend | 423107 | Live/Dead Zombie UV™ | Zombie UV™ |
| Biolegend | 304035 | CD45 | BV510 |
| Biolegend | 351321 | CD127(IL-7Rα) | PerCP/Cyanine5.5 |
| Biolegend | 350117 | CD294 (CRTH2) | PE/Cyanine 7 |
| Biolegend | 313229 | CD117(c-kit) | BV711 |
| Biolegend | 343503 | CD34 | FITC |
| Biolegend | 306013 | CD123 | FITC |
| Biolegend | 334607 | FcεRIα | FITC |
| Biolegend | 301603 | CD11c | FITC |
| Biolegend | 348801 | Lineage Cocktail^*^ | FITC |

^*^: This anti-Human Lineage Cocktail is composed of antibodies for CD3, CD14, CD16, CD19, CD20, and CD56.
